# Effect of Hypotensive Resuscitation with a Novel Combination of Fluids in a Rabbit Model of Uncontrolled Hemorrhagic Shock

**DOI:** 10.1371/journal.pone.0066916

**Published:** 2013-06-21

**Authors:** Yu-ming Zhang, Bo Gao, Juan-juan Wang, Xu-de Sun, Xi-wen Liu

**Affiliations:** 1 Department of Anesthesiology, Tangdu Hospital, Fourth Military Medical University, Xi’an, P. R. China; 2 Department of Surgery, the Affiliated Hospital of Yan’an University, Yan’an, P. R. China; 3 Department of Orthopedic Surgery, Xijing Hospital, Fourth Military Medical University, Xi’an, P. R. China; 4 Department of Nursing, Fourth Military Medical University, Xi’an, P. R. China; University of Leicester, United Kingdom

## Abstract

**Objective:**

The aim of this study was to compare the effects of hypotensive and normotensive resuscitation with a novel combination of fluids via lactate Ringer’s solution (LRS), 6% hydroxyethyl starch 130/0.4 solution (HES), and 7.5% hypertonic saline solution (HSS) at early stage of uncontrolled hemorrhagic shock (UHS) before hemostasis.

**Methods:**

New Zealand white rabbits (n = 32) underwent UHS by transecting the splenic parenchyma, followed by blood withdrawal via the femoral artery to target mean arterial pressure (MAP) of 40–45 mmHg. Animals were distributed randomly into 4 groups (n = 8): in group Sham, sham operation was performed; in group HS, UHS was untreated; in group HS-HR, UHS was treated by hypotensive resuscitation with HSS and LRS+HES (ratio of 2∶1) to MAP of 50–55 mmHg; in group HS-NR, UHS was treated by normotensive resuscitation with HSS and LRS+HES (ratio of 2∶1) to MAP of 75–80 mmHg. Outcomes of hemodynamics, inflammatory and oxidative response, and other metabolic variables were measured and the histopathological studies of heart, lung and kidney were performed at the end of resusucitation.

**Results:**

Hypotensive resuscitation with the novel combination of fluids for UHS rabbits decreased blood loss, maintained better stabilization of hemodynamics, and resulted in relatively higher hematocrit and platelet count, superior outcomes of blood gas, and lower plasma lactate concentration. Besides, hypotensive resuscitation attenuated the inflammatory and oxidative response significantly in UHS rabbits.

**Conclusion:**

Hypotensive resuscitation with the novel combination of fluids via HSS and LRS+HES (ratio of 2∶1) has an effective treatment at early stage of UHS before hemostasis.

## Introduction

Traumatic hemorrhagic shock remains a major cause of disability and death due to a life-threatening loss of blood. Most cases of traumatic hemorrhagic shock are uncontrolled hemorrhagic shock (UHS) [1]. Various strategies of ﬂuid resuscitation have been developed in response to UHS before hemostasis. Normotensive resuscitation is to use large volume of fluids to restore normal blood pressure. This method can recover blood pressure rapidly, but may cause severe hemodilution, and coagulation disturbance, resulting in an increase in blood loss and mortality [2]. Thus, the concept of hypotensive resuscitation has been raised decades before hemostasis. Hypotensive resuscitation involves resuscitating the patients to mean arterial pressure (MAP) of approximately 50 mmHg or systolic pressure of 80–90 mmHg rather than aiming for normal blood pressure [3]. In the short term, this strategy can be successful in maintaining desired perfusion while not worsening blood loss. However, no consensus has yet been reached on the best strategies of fluid resuscitation at the early stage of UHS.

There has been vigorous debate on optimal selection of resuscitation fluids. From crystalloids, to colloids, to hypertonic solutions, to artificial oxygen carriers, to blood substitutes, even to pharmacologic agents [4], [5], it is still far from a clear conclusive recommendation on the types of fluids. Lactated Ringer’s solution (LRS) is suggested by most current guidelines as the initial step in the treatment of hemorrhage shock. Studies [6], [7] showed that LRS offered a physiologic buffer against acidosis and led to a reduction in systemic inﬂammation compared with resuscitation with saline. Recently, colloids are advocated in UHS as they are associated with rapid attainment of circulatory goals, and 6% hydroxyethyl starch 130/0.4 solution (HES) is one of them. HES is a moderate resuscitation fluid with minimal effects on coagulation, and thought to be an effective volume expander and relatively innocuous. Previous researches [8], [9] suggested that HES prevented the early inﬂammatory response and oxidative stress after hemorrhagic shock and resuscitation in rats, and HES was not associated with increased bleeding. Especially, in a laboratorial study [10], in rats, organ function and survival time after UHS were improved significantly when resuscitation target MAP of 50–60 mmHg was applied with infusion of LRS and HES at a ratio of 2∶1. Another new development has focused on 7.5% hypertonic saline solution (HSS), not just as a volume expander but as an immune-response modulator. Researchers [11] reported that HSS could mobilize interstitial fluids into the vascular space, and then expand plasma volume after rapid blood loss. In addition, studies [12], [13] demonstrated that HSS might have beneficial effects by modulating the excessive immune-inflammatory response on hemorrhagic shock. Particularly, HSS was said to play an important role in the treatment of traumatic brain injury via raising intracranial pressure and cerebral oxygenation [14], [15]. In recent study, authors [16] found that HSS also improved hemodynamic function by stimulating cardiac sympathetic nerve activity after hemorrhage shock. However, it is unclear whether the combination of above three liquids in hypotensive or normotensive resuscitation has effects on USH or not.

Based on the beneficial effects of LRS+HES (ratio of 2∶1) and HSS, it is speculated that compared with normotensive resuscitation, hypotensive resuscitation with the novel combination of fluids may have a more effective treatment at early stage of UHS before hemostasis. To confirm the hypothesis, the combination of three fluids for resuscitation strategy was adopted in UHS rabbits, and the efficacy of fluid resuscitation was observed and compared in present study.

## Materials and Methods

### Resuscitation Fluids

Three fluids were used: lactated Ringer's solution (LRS, Sichuan Kelun Pharmaceutical Co., Ltd.), 6% hydroxyethylstarch 130/0.4 solution (HES, Chengdu Zheng Kang Pharmaceutical Co., Ltd.), 7.5% hypertonic saline solution (HSS, Pharmacy Center of the Fourth Military Medical University).

### Animals Management

Thirty-two New Zealand white rabbits weighing 3.0 kg to 4.0 kg were housed in a controlled environment and allowed access to chow and water ad libitum during a 7-d adaptation period before being used in this study. Twelve hours before the experiment, rabbits were fasted but allowed water ad libitum. This study was carried out in strict accordance with the recommendations of the Guide for the Care and Use of Laboratory Animals by the National Institutes of Health. The protocol of animal experiment was approved by the Animal Care and Use Committee of the Fourth Military Medical University in China (Permit Number: SYXK2007-020). The individuals conducting the experimental animal production were required to treat animals humanely. The use of experimental animals was in accordance to the requirements of scientificity, and rationality. All surgery was performed under sodium pentobarbital anesthesia. Every effort was made to minimize any suffering of the animals used in this study.

### Uncontrolled Hemorrhagic Shock Model

UHS was induced by transecting the rabbit’s spleen, followed by blood withdrawal via the femoral artery to mean arterial pressure (MAP) of 40–45 mmHg. In brief, following anesthesia and blood vessel cannulation, a midline laparotomy was performed, and the splenic parenchyma was transected transversely at one location between the major branches of the splenic artery. During the operation, great care needed to be taken to avoid injury of splenic artery branches. The cut edges of the spleen were allowed to bleed freely into the peritoneal cavity, and the laparotomy incision was closed with a running suture. Five min after the transecting spleen, the animal’s blood was withdrawn via the femoral artery for about 10 min until the MAP of 40–45 mmHg was reached. MAP was maintained at this level for 15 min by withdrawing additional amounts of blood or re-infusing blood which was aspirated into a sterile heparinized syringe and stored at room temperature as needed. To maintain the body temperature at 37°C, rabbits were placed on a warming plate. The experimental schedule of UHS induction referred to Yu.et al. [17].

### Experimental Protocol

**Figure 1:**
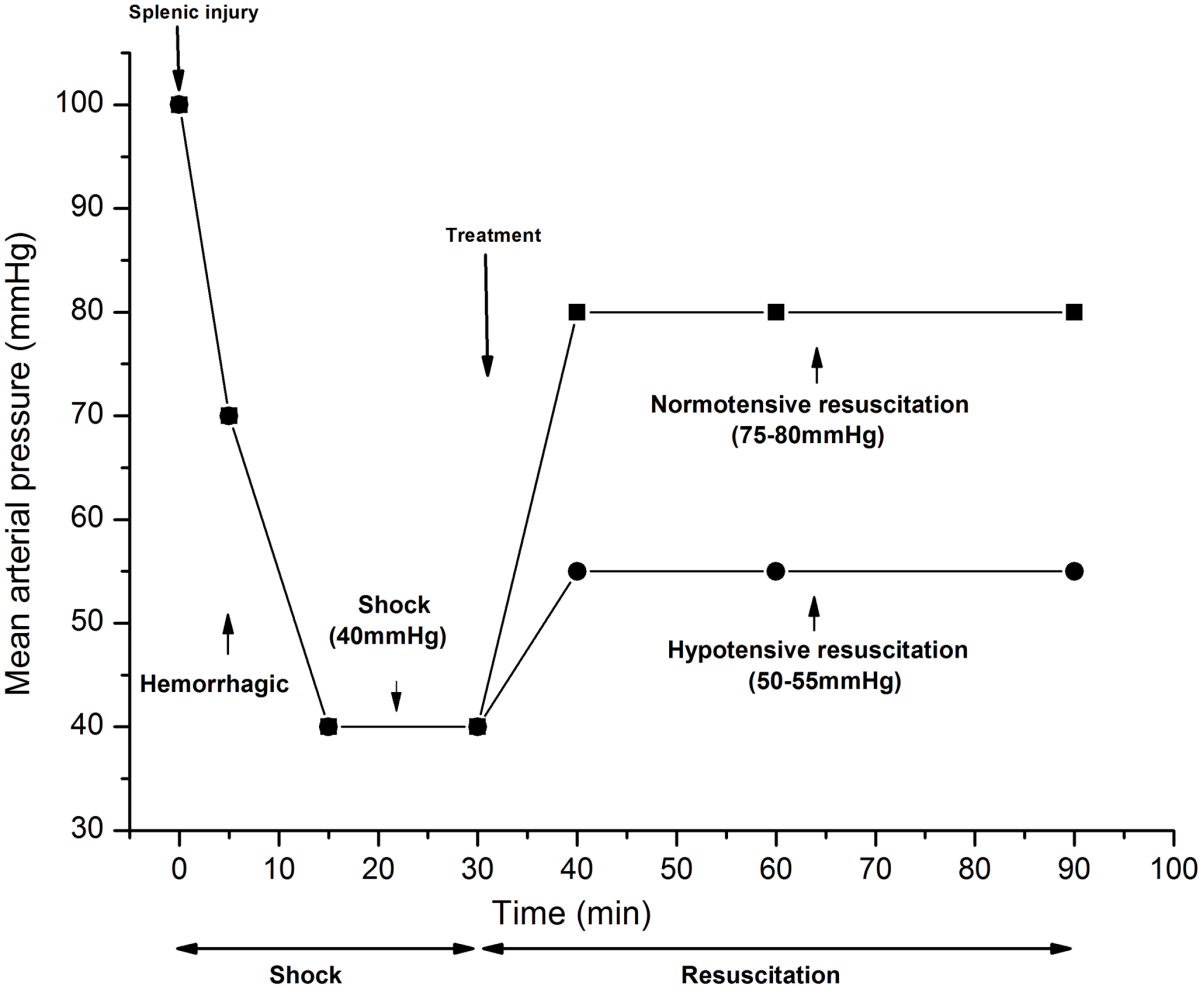
Protocol of shock and resuscitation. Rabbits were subjected to UHS by transecting spleen, followed by blood withdrawal via the femoral artery to MAP of 40–45 mmHg. MAP was maintained at 40–45 mmHg for 15 min by withdrawing or re-infusing blood. At T30, animals in group HS-HR, HS-NR were treated with a bolus of 4 ml/kg 7.5% HSS in 10 min. In group HS-HR, rabbits were treated with LRS+HES (ratio of 2∶1) to maintain MAP of 50–55 mmHg for 50 min (hypotensive resuscitation). In group HS-NR, rabbits were treated with LRS+HES (ratio of 2∶1) to maintain MAP of 75–80 mmHg for 50 min (normotensive resuscitation).

On the day of the experiment, animals were anesthetized by ear vein intravenous injection of sodium pentobarbital (30 mg/kg). Animals were kept supine and spontaneously breathing during the experiments. Polyethylene catheters (PE-50, Intramedic Medical Formulation, Clay-Adams, Parsippany, NJ) were introduced into right femoral artery and left and right femoral vein for bleeding, monitoring MAP, collecting blood samples and infusing fluids. Left ventricular catheterization via the right carotid artery was done for observing hemodynamics. The heparinized (50 U/ml) arterial line containing a calibrated pressure transducer, was directly connected to a data-acquisition system (BL-420F, Chengdu Taimeng Scientific Techonology, Inc.) After anesthesia and blood vessel cannulation, rabbits (n = 32, 8/group) were distributed randomly: in group Sham, sham operation was performed; in group HS, UHS was untreated; in group HS-HR, UHS was treated by hypotensive resuscitation with HSS (4 ml/kg) and LRS+HES (ratio of 2∶1) to MAP of 50–55 mmHg; in group HS-NR, UHS was treated by normotensive resuscitation with HSS (4 ml/kg) and LRS+HES (ratio of 2∶1) to MAP of 75–80 mmHg (Fig. 1).

Blood samples for hematocrit (Hct), platelet count, base excess (BE), PaCO_2_ (partial pressure of CO_2_ in arterial blood), PaO_2_ (partial pressure of O_2_ in arterial blood), blood pH, plasma lactate concentration, superoxide dismutase (SOD), malondialdehyde (MDA), interleukin-6 (IL-6), tumor necrosis factor-α (TNF-α) were taken at T0 (baseline), T30, T60, T90. The volume of each blood sample was immediately replaced with an equal volume of saline solution. At T90, blood loss was collected with pre-weighed pieces of dry cotton in abdomen cavity and measured according to the weight of cotton. The rabbits were sacrificed with obtaining the pathological section of heart, lung and kidney to observe the injury degree.

### Systemic Parameters Measurements and Blood Analysis

Left intraventricularsystolic pressure (LVSP), maximal change rate in left intraventricular pressure (±dp/dt max) were monitored continuously and obtained directly from the data-acquisition system (BL-420F) at T0, T30, T60 and T90. Hct and platelet count were determined with Blood Analyzer (XE2100; Sysmex, Japan). BE, blood pH, PaCO_2_, and PaO_2_ were determined with a Blood Gas Analyzer (Premier 3000, Instrumentation Laboratory, America). Plasma lactate concentration was measured with a test kit (Nanjing Jiancheng Bioengineering Institute, China). SOD, MDA, IL-6 and TNF-α were detected by a test kit (Nanjing Jiancheng Bioengineering Institute, China) and spectrophotometry (Sigma Diagnostics. Kiryat Weitzman, Rchovot, Israel).

### Pathological Section

At the end of the resuscitation, rabbits were sacrificed under an overdose of sodium pentobarbital anesthesia. The heart, lung and kidney were removed; they were fixed in 10% buffered formalin, sectioned, and stained with hematoxylin and eosin (H&E) for microscopic examination.

### Statistical Analysis

Results were presented as mean ± standard deviation (SD) unless otherwise noted, and analyzed by multivariate ANOVA with repeated measures and covariance design was used to determine if a variable changed significantly with respect to time. This test was performed after homogeneity of variances determined. When significant differences between groups were found, multiple comparison tests (Student-Newman-Keuls, SNK-q, P<0.05 was considered statistically significant) were used. Analysis was completed with a statistical software package for desktop computers (SPSS 13.0).

## Results

According to the data-acquisition system, the range of normal MAP in rabbits was between 76.29 mmHg and 95.79 mmHg.

**Figure 2:**
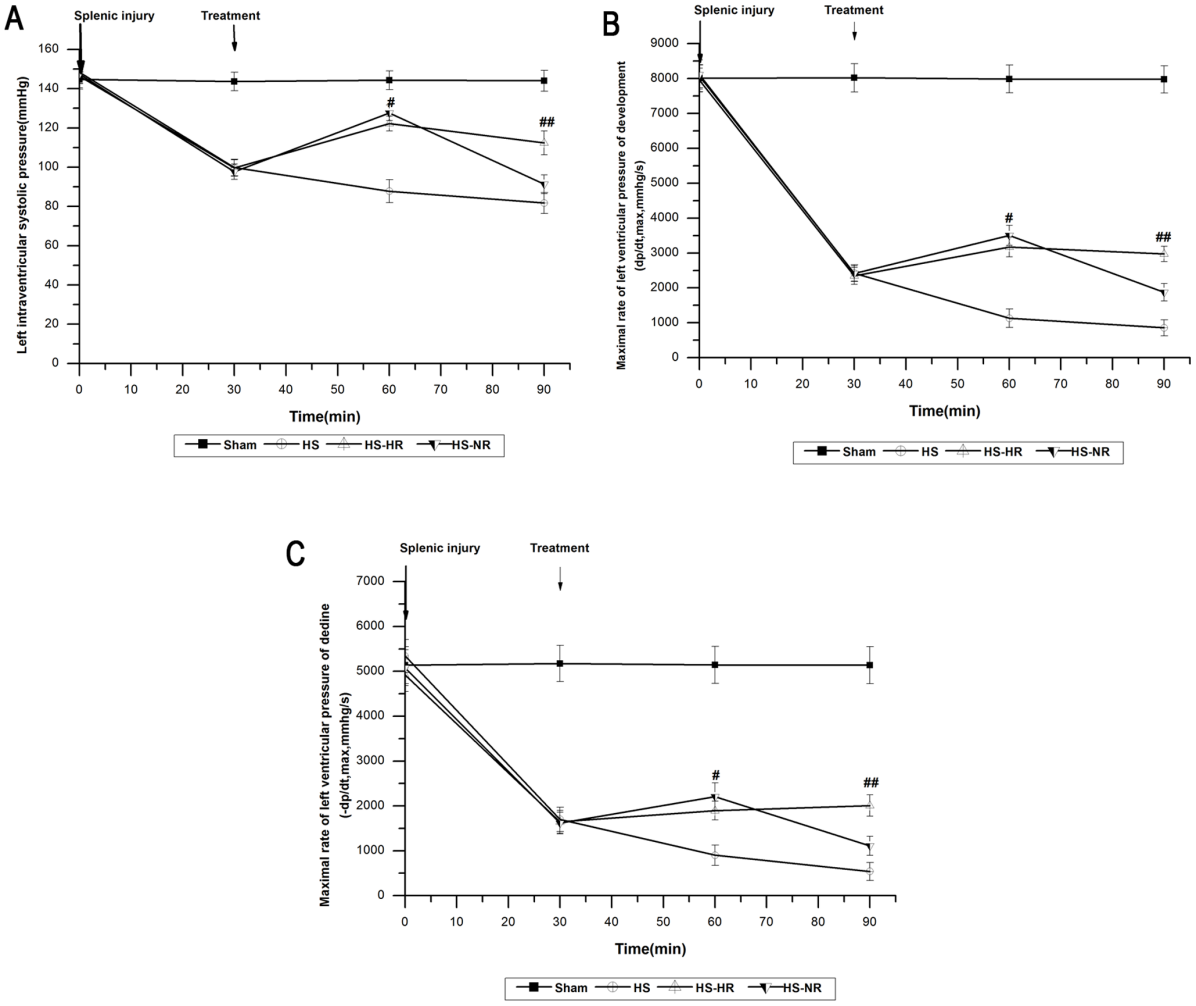
Hemodynamic parameters at T0, T30,T60, and T90. #P>0.05: At T60, LVSP and ±dp/dt max were not significantly different between group HS-HR and group HS-NR. ##P<0.001:At T90, LVSP and ±dp/dt max in group HS-HR were significantly higher than those in group HS-NR. Group Sham: sham operation; Group HS: shock with untreated; Group HS-HR: hypotensive resuscitation; Group HS-NR: normotensive resuscitation. n = 8 for all groups.

**Figure 3:**
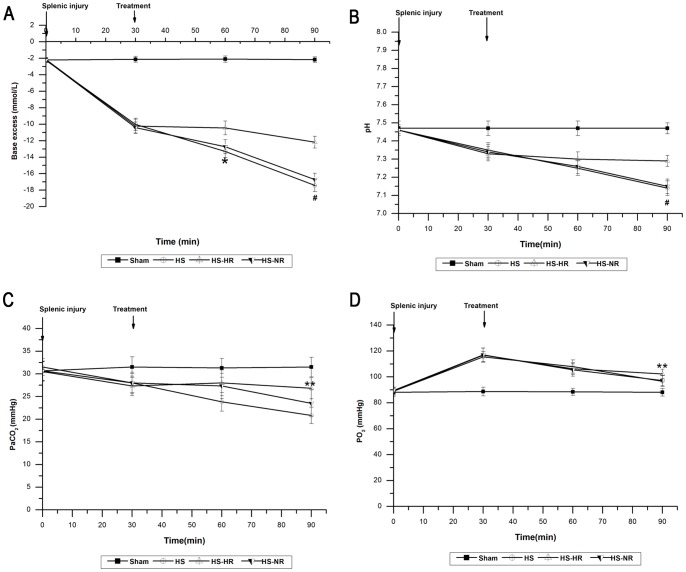
Blood gas parameters at T0, T30,T60, and T90. *P<0.001: At T60, BE in group HS-HR was significantly higher than that in group HS-NR. #P<0.001: At T90, BE and blood pH in group HS-HR were significantly higher than those in group HS-NR. **P<0.02: At T90, PaCO_2_ and PaO_2_ in group HS-HR were significantly higher than those in group HS-NR. Group Sham: sham operation; Group HS: shock with untreated; Group HS-HR: hypotensive resuscitation; Group HS-NR: normotensive resuscitation. n = 8 for all groups.

**Figure 4:**
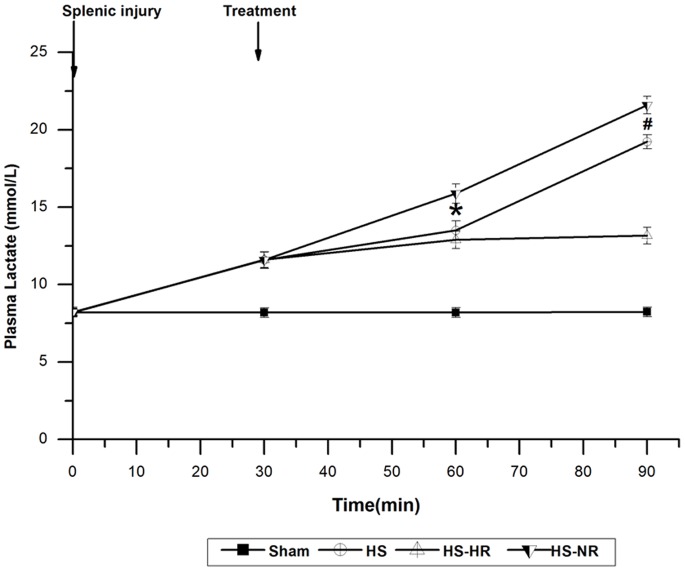
Plasma lactate at T0, T30, T60, and T90. *P<0.001: group HS-HR vs. group HS-NR. #P<0.001: group HS-HR vs. group HS-NR. Group Sham: sham operation; Group HS: shock with untreated; Group HS-HR: hypotensive resuscitation; Group HS-NR: normotensive resuscitation. n = 8 for all groups.

**Figure 5:**
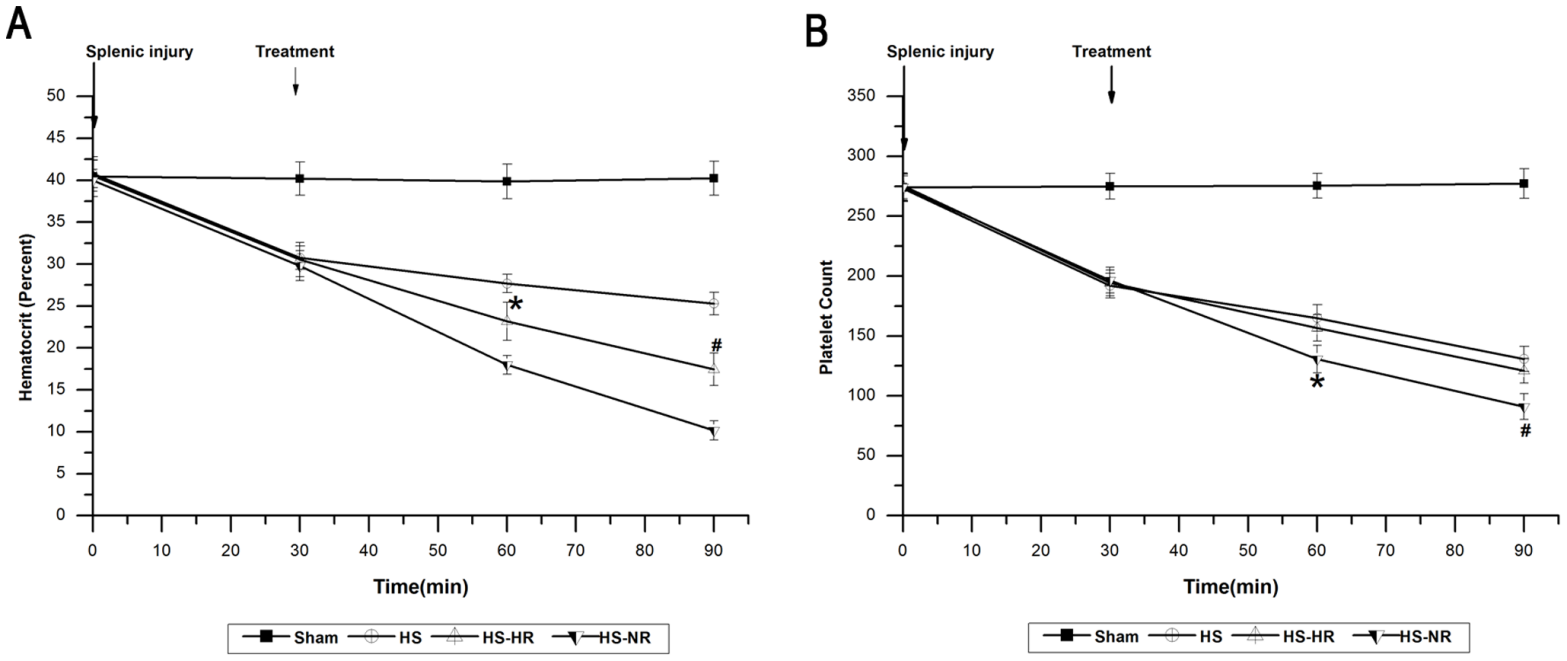
Hct and platelet count at T0, T30,T60, and T90. *P<0.001: group HS-HR vs. group HS-NR. #P<0.001: group HS-HR vs. group HS-NR. Group Sham: sham operation; Group HS: shock with untreated; Group HS-HR: hypotensive resuscitation; Group HS-NR: normotensive resuscitation. n = 8 for all groups.

**Figure 6:**
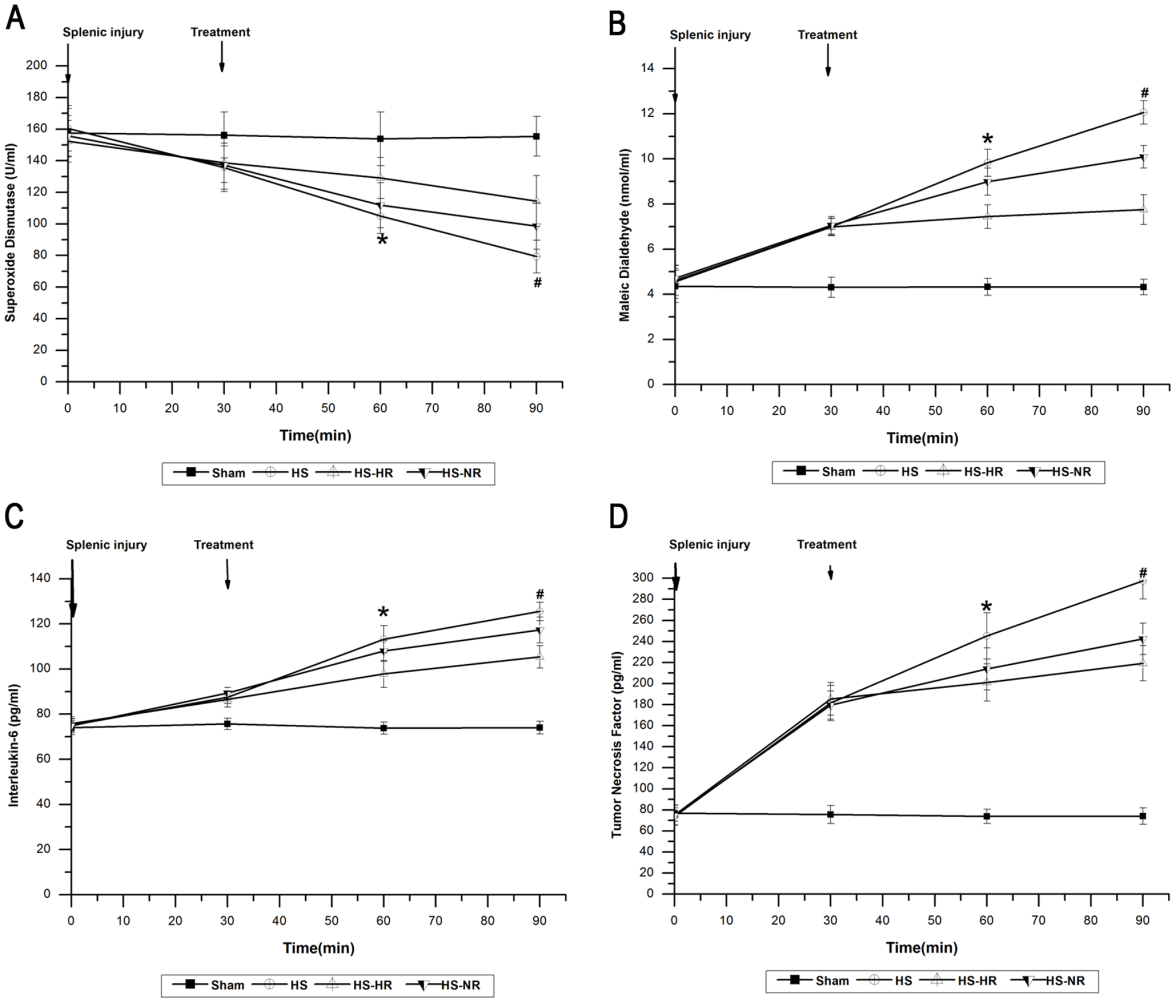
SOD, MDA, IL-6, and TNF-α at T0, T30,T60, and T90. *P<0.05: At T60, MDA, IL-6 and TNF-α in group HS-HR were significantly lower than those in group HS-NR. At T60, SOD in group HS-HR was significantly higher than that in group HS-NR. #P<0.05: At T90, MDA, IL-6 and TNF-α in group HS-HR were significantly lower than those in group HS-NR. At T90, SOD in group HS-HR was significantly higher than that in group HS-NR. Group Sham: sham operation; Group HS: shock with untreated; Group HS-HR: hypotensive resuscitation; Group HS-NR: normotensive resuscitation. n = 8 for all groups.

The hemodynamics, Hct, platelet count, blood gas, plasma lactate concentration, SOD, MDA, IL-6, and TNF-α in group Sham were stable throughout the experiment (Figs. 2–6).

### Hemodynamics Parameters

Parameters in group HS had a significant fall in LVSP from 148.43±5.78 to 99.79±4.26 mmHg Fig. 2A); in +dp/dt max from 8065.88±329.37 to 2423.36±236.37 mmHg/s (Fig. 2B), and in –dp/dt max from 5342.16±369.03 to 1700.15±269.43 mmHg/s (Fig. 2C) at T30. A similar fall in LVSP and ±dp/dt max was observed in groups HS-HR, HS-NR (Fig. 2). At T60, LVSP and ±dp/dt max showed no significant differences between group HS-HR and group HS-NR (Fig. 2). At T90, LVSP and ±dp/dt max in group HS-HR were significantly higher than those in group HS-NR (P<0.001; Fig. 2).

### Blood Gas, Plasma Lactate Concentration, Hct and Platelet Count

The blood gas parameters changes were shown in Fig. 3. During the period (T0–T30), in group HS, BE decreased from −2.25±0.29 to −10.03±0.74 mmol/L (Fig. 3A); blood pH decreased from 7.46±0.02 to 7.35±0.04 (Fig. 3B); PaCO_2_ decreased from 31.50±1.87 to 28.00±2.10 mmHg (Fig. 3C), and PaO_2_ increased from 89.00±3.16 to 115.50±4.18 (Fig. 3D). Similar changes in BE, blood pH, PaCO_2_ and PaO_2_ were observed in groups HS-HR, HS-NR (Fig. 3). At T60, BE in group HS-HR was significantly higher than that in group HS-NR (P<0.001; Fig. 3A), and blood PH, PaCO_2_ and PaO_2_ showed no significant differences between group HS-HR and group HS-NR. At T90, BE and blood pH in group HS-HR were significantly higher than those in group HS-NR (P<0.001; Fig. 3A, B). At T90, PaCO_2_ and PaO_2_ in group HS-HR were significantly higher than those in group HS-NR (P<0.02; Fig. 3C, D).

Plasma lactate concentration in group HS increased from 8.19±0.23 to 19.23±0.45 mmol/L (Fig. 4). Plasma lactate concentration in group HS-HR was significantly lower than that in group HS-NR at T60 (P<0.001) and T90 (P<0.001).

Hct in group HS decreased from 40.77±1.66 to 25.30±1.35% (Fig. 5A), and platelet count in group HS decreased from 273.00±10.86 to 130.83±10.46 ×10^9^ during the period T0–T90. (Fig. 5B). Hct and platelet count in group HS-HR were significantly higher than those in group HS-NR at T60 (P<0.001) and T90 (P<0.001).

### SOD, MDA, IL-6, and TNF-α

During the period (T0–T90), in group HS, SOD decreased from 157.43±15.17 to 79.32±10.31 U/mL (Fig. 6A); MDA increased from 4.32±0.58 to 12.06±0.52 nmol/mL (Fig. 6B); IL-6 increased from 74.93±2.66 to 125.53±4.09 pg/mL (Fig. 6C);

TNF-α increased from 73.78±8.24 to 297.53±17.09 pg/Ml. (Fig. 6D) MDA, IL-6 and TNF-α in group HS-HR were significantly lower than those in group HS-NR at T60 and T90. (P<0.05; Fig. 6B, C, D) SOD in group HS-HR was significantly higher than that in group HS-NR at T60 and T90. (P<0.05; Fig. 6A).

### Histopathologic Changes of Heart, Lung and Kidney

**Figure 7:**
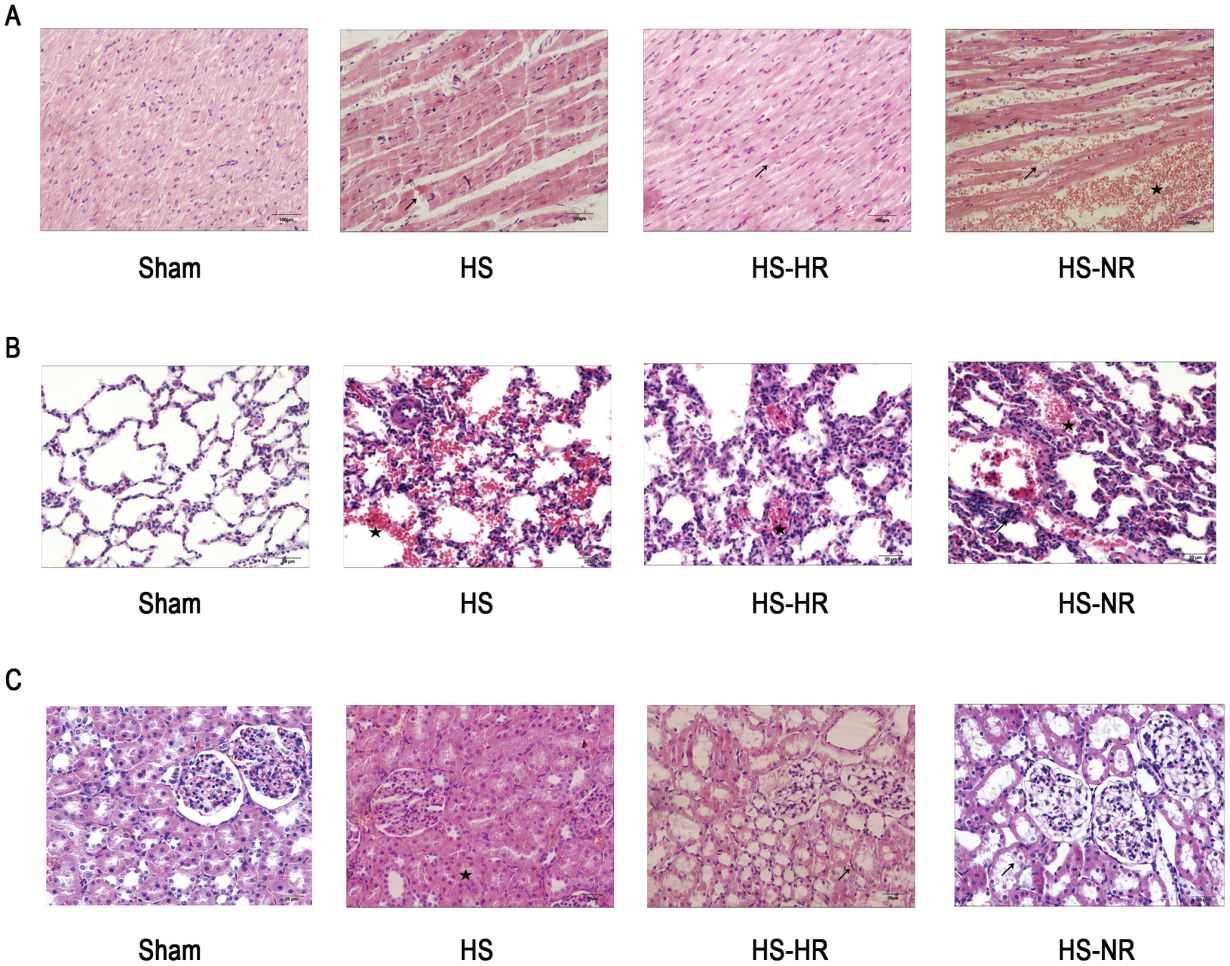
Histopathologic changes of heart, lung and kidney after shock and resuscitation. Representative pictures of three organs of animals are shown. (A): photomicrographs of heart; (B): photomicrographs of lung; (C): photomicrographs of kidney. Group Sham: sham operation; Group HS: shock with untreated; Group HS-HR: hypotensive resuscitation; Group HS-NR: normotensive resuscitation. n = 8 for all groups.

The histopathologic changes of organs were shown in Fig. 7A. B. C.

No obvious histopathologic alterations were observed in group Sham.

**Heart** (Fig. 7A) In group HS: Cardiac muscle fibers were loosely arranged, and broken (arrow), with scattered red blood cells and some infiltrated inflammatory cells. In group HS-HR: Myocardial fibers were arranged in order (arrow), with scattered inflammatory cells. No obvious changes were detected. In group HS-NR: The myocardial fibers were disrupted by a prominent intersitial infitrate of red blood cells (the star shape). Cardiac muscle fibers were loosely arranged. The broken fibers could be detected (arrow), and inflammatory cell infiltration could be observed.

**Lung** (Fig. 7B) In group HS: Histopathologic changes of the lungs showed edema, congestion, and thickened alveolar wall. In alveolus, angiotelectasis and hemorrhage were observed. Almost all alveoli were packed with visible red cells and occasional macrophages (the star shape). In group HS-HR: In alveolus, angiotelectasis and hemorrhage were still observed (the star shape). The structural integrity was not broken, showing slightly thickened alveolar walls. In group HS-NR: The structural integrity was not broken. Thickened alveolar septum (arrow) was observed obviously, and slight neutrophil infiltration in the alveoli was observed. Besides, spilled fluids in some alveoli could be detected.

**Kidney** (Fig. 7C) In group HS: The structure of glomerulus was basically normal, only with swollen renal tubular epithelial cells, the narrowed lumen (the star shape) and slight congestion in renal blood vessel. In group HS-HR: The structural integrity was not broken, showing slight renal vascular congestion. The lack of significant intersitial inflammation (arrow) was detected. In group HS-NR: The structural integrity was basically not destroyed, showing local swollen renal tubular epithelial cells or even vacuolation of renal tubular epithelial cells(arrow), Intersitial inflammation is minimal.

### Blood Loss, Volume Infusion

The blood loss in group HS-HR (53.17±15.45 mL) was less than that in group HS-NR (78.67±15.59 mL) (P<0.05). It showed no significant difference between group HS-HR and HS (45.41±9.20 mL). Infused volume of LRS+HES required to reach target MAP in group HS-HR (LRS: 56.50±10.19 mL; HES: 27.17±6.81 mL) was significantly lower than that in group HS-NR (LRS: 111.47±10.26 mL; HES: 55.60±3.67 mL) (P<0.05).(Table 1).

**Table 1:** Blood loss and infused volume.

	Blood loss(mL)	Infused volume (mL)	
Group Sham	–	LRS -	HES -
Group HS	45.41±9.20	–	–
Group HS-HR	53.17±15.45*	56.50±10.19	27.17±6.81
Group HS-NR	78.67±15.59	111.47±10.26	55.60±3.67

* P<0.05: group HS-HR vs. group HS-NR. Group HS: shock with untreated; Group HS-HR: hypotensive resuscitation; Group HS-NR: normotensive resuscitation. n = 8 for all groups.

## Discussion

A great deal of laboratory and clinical researches have demonstrated that normotensive resuscitation causes severe dilution of important hemostatic modulators, deteriorates acidosis, worsens blood loss, and then increases mortality in UHS [18], [19], [20], [21], [22]. Thus, the beneficial effects of hypotensive resuscitation at early stage of UHS before hemostasis have been advocated. In additional, various resuscitation fluids also have different impact on hemorrhagic shock. However, the ideal resuscitation strategy and resuscitation fluids for UHS remain poorly defined.

The current study investigated the effects of the normotensive and hypotensive resuscitation with a novel combination of fluids via HSS and LRS+HES (ratio of 2∶1) at early stage of UHS before hemostasis. These findings suggested that compared with normotensive resuscitation, hypotensive resuscitation with the new combination of fluids decreased blood loss in UHS rabbits, and contributed to better stabilization of hemodynamics. Other significant outcomes of this study were that with the combination of fluids, hypotensive resuscitation led to relatively higher hematocrit, superior outcomes of blood gas, lower lactate concentration, especially, attenuation of inflammatory and oxidative response in UHS rabbits. The obvious effects of hypotensive resuscitation with proper fluids combination on UHS may be closely related to the decrease of traumatic coagulopathy, the improvement of mitochondrial function and the perfusion of vital organs of rabbits.

The criteria for the selection of model must not only meet the need for experimental control but also adequately reflect the clinical pathophysiology of shock. Traditionally, UHS model is usually established by blunt trauma of solid-organ (liver or spleen) injury, or/and by large-vessel injury [23], [24]. Solid-organ injury combined with large-vessel injury can mimic UHS, but the model is too difficult to reproduce because of severe impairment of tissue [25]. Moreover, previous study concluded that outcomes from single large-vessel injury models were not applicable to other cases of injuries and that the results from clinical studies of penetrating torso trauma were not in consistent with those of blunt trauma of solid-organ injury [24], [26]. In present study, UHS was induced via spleen injury by transection of splenic parenchyma between the major branches of the splenic artery [24]. The Advanced Trauma Life Support (ATLS) demonstrates that pre-hospital fluid resuscitation in the first “golden hour” may be crucial for survival. Then, in current study, the time point of resuscitation was under this recommendation. Furthermore, there were no deaths during the hemorrhage and resuscitation period, showing the good stability and repeatability of the model. These findings suggest that the model of UHS is ideally appropriate to present study.

In previous study, Li et al. [10] found that appropriate hypotensive resuscitation target MAP of 50–60 mmHg might have the beneficial effects on UHS. And their findings showed that hypotensive resuscitation resulted in good outcomes: stable hemodynamics, decreased blood loss, good animal survival, good organ function, acid-base balance, etc. In present study, we determined the target MAP of 50–55 mmHg in hypotensive resuscitation. Our current outcomes were also similar to those of Li et al. Apart from the routine parameters, we also gathered some special index to elucidate the effective treatment of hypotensive resuscitation. Plasma lactate and BE [27], [28], [29], [30] are the most satisfactory biomarker of resuscitation, and considered as sensitive indicators of efficacy of volume expansion. Some laboratorial and clinical studies use them as the resuscitation endpoint due to the effect of reflecting the systemic tissue perfusion completely. In present study, we got the relatively ideal outcomes of plasma lactate concentration and BE at end of hypotensive resuscitation, suggesting that hypotensive resuscitation had superior effect on systemic tissue perfusion in UHS during the process of fluid infusion.

Traumatic coagulopathy is a hypocoagulable state which occurs after severe injury or exacerbated bleeding. The case of traumatic coagulopathy often results from a variety of independent but interacting mechanisms [31]. Hemodilution is one important mechanism among them. Hemodilution induced by massive fluid infusion could worsen shock-induced hypocoagulation [32]. Morrison et.al [3] demonstrated hypotensive resuscitation with small volume fluids targeting MAP of 50 mmHg resulted in less postoperative coagulopathy, and fewer plasma transfusions, and reduced intraoperative blood transfusion. In present study, we chose Hct and platelet count as the predictions of traumatic coagulopathy. Our findings were consistent with those of Morrison et al. Compared with normotensive resuscitation, hypotensive resuscitation decreased blood loss obviously, and resulted in relatively higher Hct and platelet count leading to permissive hemodilution. However, other key predictions of traumatic coagulopathy (e.g. PT, APTT, PT-INR) were not involved, so whether our resuscitation strategy may influence them needs to be explored.

The focus of our current study was to determine the effect of the resuscitation strategy with the new combination of fluids on systemic inflammatory and oxidative response at the end of resuscitation. In a sense, resuscitation is a reperfusion process and may cause mitochondrial dysfunction in cells. Then, SOD is suppressed, companying with the poor ability of scavenging oxygen radicals. Excessive oxygen radicals may induce a chain lipid peroxidation reaction and generate MDA. The combined measure of SOD and MDA can better reflect the degree of oxidative response. In addition, IL-6 and TNF-α [8], [33], [34], [35], [36] are mediators of organ injury or dysfunction and are considered as sensitive biomarkers of inflammatory response. Since the four variables could reasonably reflect the systemic immune response, we chose them to elucidate the effect of resuscitation strategy in the present study. The data showed that hypotensive resuscitation attenuated the inflammatory and oxidative response significantly. The main reason may be linked to the beneficial effect of HSS. A large number of previous studies demonstrated that HSS has the potential to modulate the excessive immune-inflammatory and oxidative response, with an overall attenuation of systemic immune mediated cellular injury [13], [37], [38], [39]. However, it should be answered why the four indices changed dramatically between normotensive and hypotensive resuscitation. The probable cause for the noted difference may be related to the effect of low plasma HSS concentration resulted from excessive dilution with large volume fluids infusion during the normotensive resuscitation. But the precise mechanism needs further investigation.

Several limitations in current study should be discussed. First, the UHS model is not real “uncontrolled hemorrhage”, because blood was withdrawn in short time to make model successful; whether this model could fully reflect UHS in humans, and whether the resuscitation strategy with the novel combination of fluids is suitable for humans needs further study. Nevertheless, this model cannot mimic the case resulted from traumatic brain injury (TBI), so our resuscitation strategy used in TBI should also be under extensive exploration. Second, the anesthetic regimen used in our study may affect the hemodynamics. However, the sodium pentobarbital only has little effect on hemodynamics [40]. Third, the data will be more persuasive with larger sample size. Fourth, due to the histopathological studies of organs, the rabbits were sacrificed at the end of resuscitation, and whether the combination of fluids could have better survival time is not examined. Fifth, it must be acknowledged that the rabbits we used in study are healthy and young, and whether the parameters are suitable for elderly patients or other cases remains to be clarified.

In summary, the current data demonstrated that normotensive resuscitation with the novel combination of fluids via HSS and LRS+HES for USH contributed to suboptimal results. Nevertheless, with the novel combination of fluids, hypotensive resuscitation resulted in superior outcomes in terms of stabilization of hemodynamics, reduction of blood loss, improvement of other variables, and attenuation of inflammatory and oxidative response at early stage of UHS before hemostasis. Whether these data could be applicable to clinical utility needs further confirmation and investigation.
